# The burden of skin and soft tissue, bone and joint infections in an Australian cohort of people who inject drugs

**DOI:** 10.1186/s12879-024-09143-0

**Published:** 2024-03-07

**Authors:** B. Morgan, R. Lancaster, B. Boyagoda, R. Ananda, LO Attwood, D. Jacka, I. Woolley

**Affiliations:** 1 Department of Medicine, Monash Health, Clayton, Australia; 2 Addiction Medicine Unit, Monash Health, Clayton, Australia; 3 Central Clinical School, Monash University, Clayton, Australia; 4 Department of Infectious Diseases, Alfred Health, Clayton, Australia; 5 Monash Infectious Diseases, Monash Health, Clayton, Australia; 6 School of Clinical Sciences, Monash University, Clayton, Australia

**Keywords:** Skin, soft tissue infections, Bone and joint infections, People who inject drugs, Addiction medicine

## Abstract

**Introduction:**

There are currently limited data regarding the clinical and economic significance of skin and soft tissue infections (SSTI) and bone and joint infections in Australian people who inject drugs (PWID).

**Methods:**

Retrospective cohort study in adult PWID admitted to Monash Health, a large heath care network with six hospitals in Victoria, Australia. Inpatients were identified using administrative datasets and International Classification of Disease (ICD-10) coding for specific infection-related conditions. Cost analysis was based on mean ward, intensive care and hospital-in-the-home (HITH) lengths of stay. Spinal infections and endocarditis were excluded as part of previous studies.

**Results:**

A total of 185 PWID (61 female, 124 male, median age 37) meeting the study criteria were admitted to Monash Health between January 2010 and January 2021. Admitting diagnoses included 78 skin abscesses, 80 cellulitis, 17 septic arthritis, 4 osteomyelitis, 3 thrombophlebitis and 1 each of necrotising fasciitis, vasculitis and myositis. Pain (87.5%) and swelling (75.1%) were the most common presenting complaints. Opioids (67.4%) and methamphetamine (37.5%) were the most common primary drugs injected. Almost half (46.5%) of patients had concurrent active hepatitis C (HCV) infection on admission. Hepatitis B (HBV) and Human Immunodeficiency Virus (HIV) were uncommon. The most significant causative organism was methicillin-susceptible *Staphylococcus aureus* (24.9%). In 40.0% (74/185) no organism was identified. Patients required a median acute hospital stay of 5 days (2–51 days). There were 15 patients admitted to the intensive care unit (ICU) with median duration 2 days. PICC line insertion for antibiotics was required in 16.8% of patients, while 51.4% required surgical intervention. Median duration of both oral and IV antibiotic therapy was 11 days. Almost half (48.6%) of patients were enrolled in an opioid maintenance program on discharge. Average estimated expenditure was AUD $16, 528 per admission.

**Conclusion:**

Skin and soft tissue and joint infections are a major cause of morbidity for PWID. Admission to hospital provides opportunistic involvement of addiction specialty services.

**Supplementary Information:**

The online version contains supplementary material available at 10.1186/s12879-024-09143-0.

## Introduction

Injecting drug use (IDU) is a significant health issue in Australia, with a recent estimate indicating there are 118,000 PWID nationwide [1]. In 2022, the Australian Institute of Health and Welfare (AIHW) estimated that 1.5% of Australians over the age of 14 have injected drugs at least once in their lifetime. In recent years, methamphetamine (54%) has overtaken heroin (35%) as the most injected drug in Australia [2].

The negative health consequences associated with IDU are well established. PWID present to Australian emergency departments with a range of diagnoses, including but not limited to; mental health disorders, overdose, injury and infections [3]. Skin and soft tissue infection (SSTI) is reported as the most common infectious complication of IDU [4]. This includes both local and systemic bacterial and fungal infections, including cellulitis, abscess formation, osteomyelitis and septic arthritis. Complications of skin, soft tissue, bone and joint infections are common, particularly when there is a delay in seeking medical attention [5]. In addition, further haematogenous spread of pathogens from SSTI can lead to more serious infections with high morbidity and mortality, including sepsis, infective endocarditis (IE) and central nervous system (CNS) infections. Furthermore, the prevalence of blood-borne viruses (HBV/HCV/HIV) is higher compared to the broader population.

Behavioural factors such as improper skin cleaning techniques, sharing needles and repeated injecting at the same site predisposes PWID to SSTI [6]. Early intervention with source control and antibiotic therapy is important to reduce morbidity and mortality associated with these infections. Harm reduction strategies have been shown to reduce the incidence of SSTI in PWID [7]. These include the use of sterile equipment and as well as education on cleaning injecting equipment after use. Linkage with speciality addiction medicine services and opioid replacement programs has also been demonstrated to reduce hospital admissions in this population over time [8].

Comprehensive understanding of the health-related complications in PWID is imperative to improve and direct a compassionate health care response. Previously, the Infectious Disease Unit and colleagues from Addiction Medicine and other units at Monash Health has described the clinical and economic significance of IE and spinal infections in PWID [9, 10]. Both studies demonstrated a significant financial burden and high rates of morbidity and mortality in this patient cohort. Here we described similar outcomes for PWID admitted with SSTI, bone and joint infections in our hospital network.

## Methods

A retrospective chart review of adult PWID (age > 18 years) admitted to Monash Health, Victoria, Australia, between January 2010 and January 2021. Monash Health is Victoria’s largest health network, consisting of six hospitals and servicing more than 1.5 million people in the Southeast of Melbourne. Monash is a university-affiliated, tertiary referral network with specialty services including infectious diseases, psychiatry, plastic surgery, orthopaedic surgery and both general and addiction medicine.

Patients were identified from an administrative dataset provided by Monash Health’s Addiction Medicine Unit (AMU). Additional cases were identified from stored medical records using diagnosis-related codes (ICD-10)(Appendix 1). Identification of cases was performed using minimum of two ICD-10 codes. One code was required to be a substance use disorder code and the second code was disease-specific related to the presenting infection. All PWID with SSTI, bone or joint infections caused by an injecting-related injury were included in the dataset.

Patients with concomitant infective endocarditis or spinal infections were excluded as these patients have previously been investigated by our unit. Admissions of less than one day were also excluded.

Admission data collected included primary diagnosis, demographics, clinical presentation, microbiology, admission length, discharge destination and follow up. Clinical cases of SSTI, bone or joint infections were identified by a combination of radiology, microbiology and clinical suspicion based on symptoms. Admission information included both acute admission (ward, intensive care unit) data and discharge destinations data (HITH and rehabilitation).

Cost estimate analysis was based on mean hospital length-of-stay for standard imputed costs provided by the Monash Business Unit. To enable comparable cost analysis with other studies undertaken by Monash Infectious Diseases, 2019 imputed costs were used. The cost per day for a standard acute ward bed was $1312; $4812 for an ICU bed; $814 for a rehabilitation bed and $1273 for HITH.

All data was stored securely on the database, REDCap [11]. Statistical analysis was performed using the software R [12]. Ethics approval was approved by the Monash Health Human Research Ethics committee (QA/80,725/MonH-2021-287052(v1)).

## Results

### Demographics

There were 185 cases of skin, soft tissue, bone and joint infections identified at the Monash Health Network between January 2010 and January 2021 in PWID. There were 115 cases included from the Addiction Medicine PWID database. Initially, 1363 cases were identified using ICD-10 codes, however, only 70 PWID were found to meet our inclusion and exclusion criteria. This included 78 episodes of abscess (42.2%), 80 cellulitis (43.2%), 17 septic arthritis (9.2%), 4 osteomyelitis (2.2%), 3 thrombophlebitis (1.6%) and 1 each of necrotising fasciitis, vasculitis and myositis (0.5%). Due to the difficultly in retrospective case ascertainment described later in the discussion, this number does not necessarily reflect all PWID who presented to Monash Health in this time period. Baseline demographics of PWID, including their blood borne virus status and injected drug preference, are summarised in Table 1.

**Table 1:** Baseline demographics of PWID

Age, median	37
Male, n (%)	124 (67.0%)
Aboriginal and/or Torres Strait Islander (ASTI), n (%)	8 (4.3%)
Blood borne viruses, n (%)	
HIV	1 (0.5%)
Hep B sAg pos	1 (0.5%)
Hep C antibody pos Hep C antibody pos (2010–2015) Hep C antibody pos (2016–2021)	106/185 (57.3%) 47/83 (51.8%) 59/102 (57.8%)
Hep C PCR pos Hep C PCR pos (2010–2015) Hep C PCR pos (2016–2021)	88/185 (47.6%) 43/83 (51.8%) 45/102 (44.1%)
Primary drug of choice, n (%)	
Opioid	124 (67.0%)
Amphetamine type stimulant (ATS)	69 (47.6%)
Other/unknown	13 (7.0%)

### Clinical presentation

Patients presented with a range of clinical symptoms. Pain was the most common presenting complaint (88.1%), followed by swelling (75.1%) and erythema (68.7%). Less common symptoms on presentation were fever (37.8%), reduced function or range of movement (24.4%), malaise (19.5%) and purulent discharge from the site of infection (10.8%). Median duration of symptoms prior to presentation to the emergency department was 4 days (IQR 5, range 1–44 days).

Culture confirmed the clinical diagnosis in 111 (60.0%) cases. There were 15 (8.1%) cases of polymicrobial infection identified. The organism was confirmed on wound (40.5%), tissue (6.3%), fluid (12.4%) and blood samples (13.0%). Species identified from culture are summarised in Table 2, individual pathogens from polymicrobial samples are included in this table. There were 14 bacterial and 2 yeast species identified as the most likely causative pathogens for the clinical presentation. Methicillin-susceptible *Staphylococcus aureus* (MSSA) was the most common pathogen identified (24.9%), followed by methicillin-resistant *S. aureus* (MRSA) (11.4%). Five *Streptococcal species* were identified; *Streptococcus pyogenes* (9.2%) represented the most common of these. An associated bacteraemia was identified in 13.0% of patients.

**Table 2:** Organisms identified and culture source

**Organism, n (%)**	
Methicillin-susceptible *Staphylococcus aureus*	46 (24.9%)
Methicillin-resistant *Staphylococcus aureus*	21 (11.4%)
*Streptococcus pyogenes*	17 (9.2%)
*Streptococcus anginosus*	10 (5.4%)
*Streptococcus dysgalactiae*	2 (1.1%)
*Streptococcus milleri*	1 (0.5%)
*Streptococcus gordonii*	1 (0.5%)
*Serratia liquefaciens*	1 (0.5%
*Staphylococcus lugdunesis*	1 (0.5%)
*Candida albicans*	1 (0.5%)
*Candida guilliermondii*	1 (0.5%)
*Actinomyces sp.*	1 (0.5%)
*Neisseria gonorrhoeae*	1 (0.5%)
*Enterococcus faecalis*	1 (0.5%)
*Enterococcus faecium*	1 (0.5%)
*Klebsiella pneumoniae*	1 (0.5%)
Negative culture	74 (40.0%)
**Type of culture, n (%)**	
Wound	75 (40.5%)
Tissue	12 (6.5%)
Fluid	23 (12.4%)
Blood	24 (13.0%)

### Outcomes

The median acute hospital length of stay was 5 days (IQR 6, range 1–51 days). Fifteen patients (8.1%) required ICU admission with a median stay of 2 days. Surgical intervention was common with more than half (51.3%) of patients requiring surgery. Procedures included incision and drainage, joint washout and debridement. The median duration of antibiotic therapy (both intravenous (IV) and oral) was 11 days (IQR 9 days, range 1–85). There were 22 patients (11.9%) that discharged prematurely or did not complete their antibiotic therapy. There were 19 patients (10.3%) admitted to HITH for ongoing antibiotic therapy or wound care and 90 patients (48.6%) of patients were enrolled in an opioid treatment program on discharge.

### Cost estimate analysis

Table 3 summarises the total expenditure of each episode of infection in this study based on admission cost per bed per day in 2019. One episode of skin, soft tissue, bone or joint infection was estimated to cost the health system $16,528 on average.

**Table 3:** Cost analysis, total expenditure per episode SSTI, bone or joint infection

	Cost/day	Total days	Expenditure	Cost/episode
Acute ward	$1312	1740	$2,282,880	$12,340
ICU	$4812	54	$259,848	$1405
HITH	$1273	349	$444,277	$2401
Rehabilitation	$813	87	$70,731	$382
			TOTAL	**$16,528**

## Discussion

SSTIs are often described as the leading cause for presentation to the emergency department (ED) for PWID [13]. Most of these presentations are preventable and understanding the complex needs of these patients is crucial to reducing serious complications. In addition to past research conducted by the Monash Infectious Disease department [9, 10], this study further explores bone and joint infections in conjunction with SSTIs in order to add to the understanding of clinical outcomes more specifically, in an Australian context.

The methodology used in our study was chosen to align with previous studies by Low et al. and Ananda et al. [9, 10] which reported on the burden of IE and spinal infections in PWID in the same South-Eastern Victorian catchment. As with our study, staphylococcal species were the most common causative organisms, suggesting the haematogenous spread of bacteria following an injecting-related skin injury. The severity of disease was much greater in both studies, compared to our SSTI cohort. This was reflected by higher in-hospital mortality (16% of IE; 1.8% of spinal infections) and longer median hospital stays (40 days for IE; 47 days for spinal infections). The average cost of IE and spinal infections to our health service was approximately $50,000 AUD more than SSTI per episode. Patients with concomitant IE and spinal infections were not included in this study to demonstrate the isolated impact of SSTI, bone and joint infections on health outcomes in PWID within our hospital service.

As previously mentioned, our study focused only on skin, soft tissue, bone and joint infections in PWID. Skin trauma associated with injecting is most commonly the source of bacterial infections [14]. Consistent with many other studies, gram positive bacteria including staphylococcal and streptococcal species represented the majority of confirmed microbiological diagnoses [4, 15]. The 2021 AURA (antimicrobial resistance use and resistance) report suggests the prevalence of community MRSA in Victoria is 11.5%. This is reflected by our study which identified 11.4% of SSTI presentations caused by MRSA [16]. Community-acquired MRSA infections are on the rise, with many countries describing MRSA as the predominant abscess causing organism in PWID [14]. Identification of subgroups at increased risk of MRSA has important implications for guidelines for appropriate community and hospital antibiotic prescribing to prevent morbidity and mortality associated with MRSA infections. This study supports the continued need for clinicians to consider MRSA cover in PWID populations.

Gram-negative bacilli (GNB) were reported in 2.2% of infections in this study. A retrospective cohort study of infections in PWID conducted at the University of Tennessee Medical Centre, described 28% of infections associated with GNB. The study described age (> 50), recent hospitalisation and joint infections as independent risk factors associated with GNB infections [17]. Current Australian guidelines for SSTI, bone and joint infections suggest antimicrobial coverage for streptococci and MRSA in high-risk patients while awaiting cultures and sensitivity [18]. While incidence of GNB-associated infections was scarcely reported in our cohort, the consideration of alternative causative organisms including gram negative bacteria and yeast species may be required in PWID presenting with bone and joint infections or those patients requiring multiple hospital admissions.

Duration of both oral and parenteral antibiotic therapy for most skin and soft tissue infections is between 5 and 14 days, depending on the clinical response to antimicrobials and adequate source control [19]. We report a median duration of antibiotic therapy (intravenous and oral) as 11 days (IQR 9, range 1–85). Extended antibiotic duration was often due to episodes of bacteraemia and complications from the primary infection. More than half of PWID admitted to our hospital with these infections required surgical intervention which were most commonly incision and drainage of superficial skin abscesses.

Prevalence of blood borne viruses in our cohort was also in concordant with that of the Australia population. We reported only one patient living with HIV, similar to the current Australian prevalence of 1–2% of PWID [20]. HCV antibody positivity in Australia is reported to be declining, with the AIHW describing a reduction in HCV antibody positivity in PWID from 51% in 2016 to 39% in 2020 [20]. HCV seropositivity in our cohort was 57.3%, which is higher than that was reported by the AIHW in this time period. Overall, 49.6% of HCV antibody positive patients were HCV RNA positive. The proportion of HCV antibody positive patients with a positive Hep C PCR decreased by 13.7% after 2016, however, this was not statistically significant (*P* = 0.0305, fisher’s exact). This may reflect widespread availability and increased uptake of directly acting antiviral agents (DAAs) introduced in Australia in 2016, however, a larger cohort would be required to substantiate this. Our results highlight an opportunity for diagnosis and referral to outpatient infectious disease clinics to facilitate treatment of HCV and prevention of several complications known to be associated with HCV, including cirrhosis and hepatocellular carcinoma.

Injecting technique and hygiene practices are well understood risk factors associated with severe infections in PWID [21]. Our cohort data conflicted with current Australian data, reporting that opioid-based preparations were more commonly used (67.0%) than amphetamine-like stimulants (ATS) (47.6%) [20]. Route of administration, in particular subcutaneous or intramuscular injection (“skin popping”) has been independently associated with risk of infection, though this technique is very uncommon in Australia [22]. A comprehensive history on drug use is rarely gathered during clinical history taking and consequently excluded from the documentation of clinical records. We were able to identify injecting practices limited to the type of drug injected, however, were unable to identify any risk factors associated with injecting practices in our cohort. Qualitative research on PWID also suggests that these patients have a tendency to delay seeking medical attention due to negative experiences in the health system secondary to stigma, acute substance withdrawal and inadequate pain management [23]. We noted a highly variable time to seeking medical attention amongst PWID presenting with SSTI, bone and joint infections (median 4 days, range 44 days). Training of clinicians to take an appropriate drug history is essential not only to identify risk factors but also mitigate other problems faced by PWID in healthcare settings such as withdrawal and inappropriate pain management [24].

Premature discharge is also a common problem for PWID and is associated with increased risk of readmission as well as poorer long-term health outcomes. A systemic review in the United States suggested this phenomenon is frequently observed amongst PWID, occurring in as many as 25–30% of admissions [25]. Absent or suboptimal management of substance use disorders (SUD) has been described as a major contributing factor for PWID who discharge prematurely [25]. This highlights the importance of seeking early involvement of specialty addiction medicine services, where available, to facilitate optimal duration of treatment as well as linkage with outpatient services on discharge. Previous studies at Monash Health on infections in PWID reported premature discharge rates between 7 and 15% [9, 10]. Similarly, our cohort had a premature discharge rate of 10.9%. Alternative strategies, including outpatient parenteral antimicrobial therapy (OPAT) through HITH services need to be considered. Regrettably, hesitation often persists to enrol PWID into outpatient services due to perceived concerns of non-adherence, staff safety and potential misuse of peripherally inserted IV catheters with other drugs. A literature review of OPAT outcomes in PWID has suggested no comparable difference in hospital readmission and infection relapse than those without history of IDU. Adherence rates in a number of cohort studies is as high as 72–100%, again similar to the general population [26]. From a quality and safety perspective, this data provides supportive evidence against some of the therapy interfering discriminatory beliefs about PWID.

Harm minimisation strategies such as opioid replacement therapy (ORT) and clean injecting equipment has been demonstrated to reduce the risk of SSTI as well as other blood borne viruses in PWID [7, 27, 28]. In our study, 90 patients, which represents 72.6% of patients using opioid-based preparations, were enrolled in ORT on discharge. This highlights the importance of opportunistic engagement and the offer of evidence-based treatment for an individual’s opioid use disorder in the hospital setting. Consolidating evidence for pharmacological treatment of methamphetamine use is currently lacking, despite extensive investigation. A recent systematic review and meta-analysis by Naji et al., demonstrated that mirtazapine may result in small reduction in methamphetamine use in patients compared to placebo (RR = 0.81, 95% CI 0.63, 1.03) [29]. While these results look promising at the time of writing, it is not currently available in Australia for the indication of methamphetamine use. In lieu of any approved pharmacological management options, psychosocial interventions, such as contingency management, remain the principal therapies for managing methamphetamine use disorder [30]. This provides emphasis on the complex needs of PWID and the requirement for the availability of multidisciplinary services in both their hospital and outpatient care.

Currently, there are no Australian studies assessing the cost of IDU-related SSTIs. The healthcare costs for infections related to IDU is substantial in our health network. We estimate the cost per episode of SSTI, bone and joint infection to be $16,528 AUD. Cost analysis of IDU-IE in our network was estimated at $74,168AUD per episode in 2015 [10]. Additionally, spinal infections related to IDU were estimated to cost $61,557 AUD per episode in 2019 [9]. Cumulatively, infections related to IDU at Monash Health is estimated to be in the magnitude of 1.25 million AUD per annum. However, the true financial burden is likely underestimated as radiological and surgical costs were not included in the cost-estimate analysis. Appropriate prescription of antimicrobial therapy and early referral to speciality addiction services have the potential to reduce hospital re-admission and costs associated with SSTIs in PWID [31].

The size of our cohort is a relative underestimation of the true burden of SSTI, bone and joint infections amongst PWID in the Monash Health Network. Two thirds of the presentations were identified using the database supplied by our Addiction Medicine Unit. There is opportunity for a separate study to assess the barriers to referral of PWID to addiction speciality services. Retrospective data collection using ICD-10 codes to identify cases that were not referred to Addiction Medicine during their admission was additionally used to identify patients in this study. Data extraction from the health record using ICD-10 codes specific for SSTI, bone and joint infections (see appendix) revealed 21,123 episodes between January 2010 and January 2021 without an additional substance use disorder code. To be included in our study, episodes of SSTI in PWID were extracted using two ICD-10 codes; including substance use disorder and a disease-related code (see appendix). Initially, 1363 cases were identified. The ICD-10 coding system does not have a specific diagnosis for IDU. The most common reason for exclusion was because the substance use code did not reflect injecting drug behaviour but rather other substance use, namely cigarette use and alcohol consumption. Furthermore, cases of IE and spinal infections were also excluded as these presentations had previously been studied by our research group.

Collection of data retrospectively via ICD-10 codes relies heavily on appropriate documentation and allocation of appropriate coding. ICD-10 coding often reflects only the primary diagnosis of a patient admission, leaving the potential for several cases of SSTI in PWID to be excluded from the study. Failure to self-report IDU behaviours due to fear of discrimination and stigmatization likely underestimate our PWID cohort. Retrospective identification of infections in PWID using ICD-10 coding may be less challenging amongst patients who required a prolonged hospital admission. A more detailed history and identification of source may have been more likely in PWID with spinal infections or IE. A recent study by Curtis et al. describing the prevalence injecting-related infections in greater Melbourne using a prospective observational cohort identified 345 PWID admitted to hospital over a ten-year period between 2008 and 2018 (27% of total emergency presentations) [32]. Given our study excluded PWID admitted to hospital for less than one day, we believe our study describes the minimum burden of IDU-related SSTI in the South-Eastern Victorian, Monash Health catchment. However, we note that this does not reflect the number of PWID presenting to our emergency departments with uncomplicated SSTIs that do not require hospital admission.

## Conclusion

This study supplements previous research completed by our group, describing a wide range of injecting-related infections experienced by PWID in an Australian context. Compared to PWID with IE and spinal infections in the Monash Health catchment, mortality and disease severity associated with isolated skin, soft tissue, bone and joint infections is significantly lower. However, morbidity and financial burden remains a problem. Limitations with data collection and clinical history taking in PWID, particularly those admitted to hospital for a shorter time period, have likely contributed to a smaller than expected sample size of PWID with isolated SSTI in our health service.

Despite this, our study highlights the complexity of the hospital admissions and unique needs of PWID, who often require holistic multidisciplinary input. There are future opportunities to develop clinical education strategies to enrich substance-use history taking, improve awareness of acute withdrawal management and expand referral rates to addiction specialty services. These strategies can be used in conjunction with OPAT to improve the overall experiences of PWID support earlier engagement with the healthcare system. Furthermore, there remains opportunities for outpatient treatment of HCV and opioid replacement therapy, Overall, the uptake of such practices may improve the health outcomes of PWID and reduce the economic burden on the healthcare system.

### Electronic supplementary material

Below is the link to the electronic supplementary material.

Supplementary Material 1

## Data Availability

Data may be available upon request subject to availability and applicability upon request from the corresponding author.
