# Supplementary material for: The burden of skin and soft tissue, bone and joint infections in an Australian cohort of people who inject drugs
Source: BMC Infect Dis. 2024 Mar 7;24:299. doi: 10.1186/s12879-024-09143-0 (PMC10918955; doi:10.1186/s12879-024-09143-0)
Supplement: Supplementary file 1 — Supplementary Material 1 [file 12879_2024_9143_MOESM1_ESM.docx]

Appendix:

| **Diagnosis** | **ICD-10** |
| --- | --- |
| Substance use disorder | F11, F13, F14, F15, F16, F19 |
| Superficial thrombophlebitis from infection | I80 |
| Pyogenic bacterial infections of the skin and subcutaneous tissues | L02, L08 |
| Bacterial infection of unspecified site | A49 |
| Bacterial cellulitis | L03, L04 |
| Joint infection | M00 |
| Osteomyelitis | M86 |
| Necrotizing fasciitis | M72 |

**Appendix 1: International Classification of Disease (ICD)-10 codes**
